# Downregulation of PTEN Promotes Autophagy *via* Concurrent Reduction in Apoptosis in Cardiac Hypertrophy in PPAR α^−/−^ Mice

**DOI:** 10.3389/fcvm.2022.798639

**Published:** 2022-02-11

**Authors:** Ritu Kumari, Aleepta Guha Ray, Dibyanti Mukherjee, Vivek Chander, Dipak Kar, Uppulapu Shravan Kumar, Deepak Bharadwaj P.V.P., Sanjay K. Banerjee, Aditya Konar, Arun Bandyopadhyay

**Affiliations:** ^1^Cell Biology and Physiology Division, CSIR-Indian Institute of Chemical Biology, Kolkata, India; ^2^Department of Biotechnology, National Institute of Pharmaceutical Education and Research, Guwahati, India; ^3^Department of Pharmacology and Toxicology, National Institute of Pharmaceutical Education and Research, Guwahati, India

**Keywords:** cardiac hypertrophy, PPAR α, apoptosis, autophagy, PTEN

## Abstract

Cardiac hypertrophy is characterized by an increase in the size of the cardiomyocytes which is initially triggered as an adaptive response but ultimately becomes maladaptive with chronic exposure to different hypertrophic stimuli. Prolonged cardiac hypertrophy is often associated with mitochondrial dysfunctions and cardiomyocyte cell death. Peroxisome proliferator activated receptor alpha (PPAR α), which is critical for mitochondrial biogenesis and fatty acid oxidation, is down regulated in hypertrophied cardiomyocytes. Yet, the role of PPAR α in cardiomyocyte death is largely unknown. To assess the role of PPAR α in chronic hypertrophy, isoproterenol, a β-adrenergic receptor agonist was administered in PPAR α knock out (PPAR α^−/−^) mice for 2 weeks and hypertrophy associated changes in cardiac tissues were observed. Echocardiographic analysis ensured the development of cardiac hypertrophy and compromised hemodynamics in PPAR α^−/−^ mice. Proteomic analysis using high resolution mass spectrometer identified about 1,200 proteins enriched in heart tissue. Proteins were classified according to biological pathway and molecular functions. We observed an unexpected down regulation of apoptotic markers, Annexin V and p53 in hypertrophied heart tissue. Further validation revealed a significant down regulation of apoptosis regulator, PTEN, along with other apoptosis markers like p53, Caspase 9 and c-PARP. The autophagy markers Atg3, Atg5, Atg7, p62, Beclin1 and LC3 A/B were up regulated in PPAR α^−/−^ mice indicating an increase in autophagy. Similar observations were made in a high cholesterol diet fed PPAR α^−/−^mice. The results were further validated *in vitro* using NRVMs and H9C2 cell line by blocking PPAR α that resulted in enhanced autophagosome formation upon hypertrophic stimulation. The results demonstrate that in the absence of PPAR α apoptotic pathway is inhibited while autophagy is enhanced. The data suggest that PPAR α signaling might act as a molecular switch between apoptosis and autophagy thereby playing a critical role in adaptive process in cardiac hypertrophy.

## Introduction

Cardiomyocyte tends to execute an adaptive mechanism in order to survive the stress induced by various external stimuli. Cardiac hypertrophy is one such example where adaptive response helps to overcome the increased demands of the failing heart. However, prolonged hypertrophy leads to pathological conditions and eventually heart failure occurs. Cardiac hypertrophy brings about significant changes that mark extensive remodeling both at the cellular and molecular levels (1, 2). Cardiomyocyte size is increased along with extensive fibrosis, marked by reprogramming of the fetal gene expression such as atrial natriuretic peptide (ANP), brain natriuretic peptide (BNP) and reduced expression of the genes for fatty acid oxidation. Available reports demonstrated that inhibition of PPAR α and PGC1 α signaling is a prerequisite for fetal genes re-expression as well as reduced fatty acid oxidation (3, 4).

Prolonged cardiac hypertrophy leads to PPAR α mediated metabolic reprogramming resulting into reduced fatty acid oxidation (5). Pressure overload in heart in response to β-adrenergic receptors induces myocardial hypertrophy and cardiac remodeling, that is accompanied by down regulation of PPAR α leading to energy metabolic disorder (6). Under normal condition, the heart utilizes saturated fats as the primary metabolic fuel. However, in the absence of PPAR α activity, accumulation of excess fat is responsible for the metabolic switch from fatty acid oxidation toward glycolysis (7–10). High-cholesterol diet has also been directly implicated in cardiac hypertrophy since the resulting lipotoxicity stimulates compensatory mechanism as a defense toward chronic pressure overload in heart (11). Severe hypercholesterolemia as well as administration of β adrenergic receptor agonist such as isoproterenol in mice hearts has been associated with significant cardiomyocyte death and development of heart failure (7, 8, 12).

Multiple signaling molecules and pathways regulate cardiomyocyte apoptosis which might lead to heart failure (13). PTEN that is reported to be widely expressed in cardiomyocytes is activated in response to I/R injury or β-adrenergic receptor stimulation and contributes to cardiac apoptosis (14, 15). PTEN functions as a lipid phosphatase that dephosphorylates phosphatidylinositol (3,4,5)-trisphosphate (PIP3) to phosphatidylinositol (4,5)-bisphosphate (PIP2) and negatively regulates PI3K/AKT signaling pathway (16). Deletion of PTEN has been associated with enhanced Akt Phosphorylation and activation of Akt, whereas over expression of PTEN results in Akt inactivation as well as cardiomyocyte death (17). PI3K/Akt pathway mediates anti-apoptotic effects in cardiomyocytes as established by Akt downregulation following pro-apoptotic insults (17). Stimulation of Akt has been reported to protect against myocardial injury and attenuate apoptosis (18).

Many reports suggest metabolic stress dependent activation of apoptotic pathway that leads to cell death as the cellular mechanism fails to cope with the increased energy demands (19). However, an inclination toward the autophagy pathway helps in sustaining the metabolic stress by promoting cell survival while defects in autophagy could promote cell death and inflammation (19, 20).

Several studies have reported the mTOR mediated regulation of autophagy (21). Autophagy is a dynamic self-degradative process that is essential to maintain cellular homeostasis. Growing evidences have revealed association between cardiac hypertrophy as well as autophagy (22, 23). Baseline autophagy functions by removing damaged organelle aggregates and preventing cardiomyocytes from cytotoxicity (22, 23). Moderate tuning of autophagy has been reported to be beneficial for the cardiac health as it maintains cellular homeostasis and prevents cell death.

Available literatures have demonstrated that PPAR α signaling plays an important role in the transcription of various genes associated with fatty acid oxidation as well as mitochondrial biogenesis and thereby plays a pivotal role in the pathophysiology of cardiac hypertrophy.

To explore the involvement of PPAR α in cardiomyocyte adaptive mechanism, PPAR α-/- mice was used that revealed significant down regulation in apoptosis that was explained by the decrease in PTEN. Several studies have implicated the direct transcriptional regulation of PTEN *via* PPARα but the sustained down regulation of PTEN in the presence of isoproterenol that is a β-adrenergic receptor agonist, was observed in our study. Based on the findings, our aim was to understand how the absence of PPAR α influences apoptosis in hypertrophied cardiomyocytes. This might also reveal how PPAR α mediated signaling influences the adaptive mechanism of the cardiomyocytes in stress-induced hypertrophy.

## Materials and Methods

### Animal Studies

The protocol was approved by the Institutional Ethics Committee (Reference no. IICB/AEC/Meeting/Sep/2019/4) and the animals were handled in accordance with the Committee for the Purpose of Control and Supervision of Experiments on Animals (CPCSEA), Ministry of Social Justice, and Government of India (Registration no. 147/1999/CPCSEA). Male and female mPPAR α^−/−^ mice (F2 homozygotes; hybrids of Sv/129 3 C57BL/6N genetic background 3 months-old were obtained from The Jackson Laboratory (Bar Harbor, Maine, USA) and bred in the Central Animal Facility CSIR-IICB, Kolkata. Genotyping of the PPARα^−/−^ mice was done using primers flanking the region that was knocked out and has been added as Supplementary Figure 4. C57BL/6 wild-type mice and PPARα^−/−^ mice were intraperitoneally injected with 30 mg/kg/day dose of isoproterenol for 14-days. 3 months-old PPARα^−/−^ mice were divided into two groups and were fed two types of diet, normal chow diet (kcal: protein = 25%; carbohydrate = 58%; fat =17%) and high cholesterol diet (HCD, Research Diets Inc., catalog no. D12336 with 1.25% cholesterol). Mice were maintained on 12-h dark /12-h light cycle and allowed to have easy access to food and water. The number of animals (n) was at least 6 for all the different experimental conditions. At the end of the experimental duration, the animals were anesthetized by an intraperitoneal injection of 100 mg/kg ketamine with 10 mg/kg xylazine hydrochloride and sacrificed *via* cervical dislocation. The heart was collected for histological and biochemical analysis.

### Histological Staining

The heart tissues were fixed in 10% formalin, embedded in paraffin and then cut into 5 μm thin sections using microtome. Haematoxylin and Eosin (H&E) staining and Masson Trichome staining were performed to observe increase in cardiomyocyte size (morphological changes) and collagen deposition, respectively. Stained slides were visualized using light microscope.

### RNA Extraction and Reverse-Transcription

RNA was isolated using Tri Reagent solution (Sigma) according to manufacturer protocol. RNA concentration and purity (A260/A280 N 1.9) was analyzed using Nanodrop 2000. Real-time PCR was performed using Luna® Universal One-Step RTqPCR Kit (NEB) with 300 ng of RNA per reaction in 96-well optical reaction plates in AB Real Time 7500 Fast system. RT-PCR primers were designed using Primer Bank. The primer sequences are provided in Table 1.

**Table 1:** Sequences of primers used for RT-PCR.

**S. No**.	**Gene**	**Forward primer**	**Reverse primer**
1	GAPDH	TGGCCTTCCGTGTTCCTAC	GAGTTGCTGTTGAAGTCGCA
2	Acaa1a	TCTCCAGGACGTGAGGCTAAA	CGCTCAGAAATTGGGCGATG
3	HSD17	GGCTTCCTGTACTGGGTGG	CACGTTTTGCTAACTCTTCTGC
	B12		
4	ACAA2	CTGCTACGAGGTGTGTTCATC	AGCTCTGCATGACATTGCCC
5	Acox1	TAACTTCCTCACTCGAAGCCA	AGTTCCATGACCCATCTCTGTC
6	PRKA	AATGAACACTTTCAAGAGAC	CCAACTTGGAACTTGTGGGA
	G2	CCC	AT
7	Ep300	TTCAGCCAAGCGGCCTAAA	CGCCACCATTGGTTAGTCCC

### Tissue Homogenization

Heart tissue was homogenized using Teflon Homogenizer in RIPA Buffer with protease inhibitor cocktail (ProteoGuard™ EDTA-Free Protease Inhibitor Cocktail, Clonetech) and centrifuged at 16,000 g at 4°C for 20 min.

### Western Blot Analysis

Protein concentrations were determined using DC™ Protein Assay Kit (Bio-Rad). Equal amount (30 ug) protein Samples were separated on 1.5 mm 10% sodiumdodecylsulfate polyacrylamide gels and transferred onto PVDF membranes (Merck-Millipore). Membranes were blocked with 5% skimmed milk or 5% BSA (HiMedia, #TC194) and probed using the following antibodies: Anti-Opa1 (ab42364), Anti-Annexin V (8555S), Anti-c-PARP (sc-56196), Anti-Caspase 9 (9508S), Anti-p53 (2524S), Anti-Mdm2 (sc-965), Anti-Dbc1 (5857S), Anti-mTOR(sc-517464), Anti-pAkt:ser-473(sc-293125), Anti-PTEN(sc-7974), Anti-Bcl-2(sc-7382), Anti-LC3 A/B(ab128025), Anti-p62(ab91526), Anti-Beclin1 (ab55878), Anti-Atg3(3415T), Anti-Atg5(12994T), Anti-Atg7(8558T), Anti-Calpain (sc-271856), Anti-GAPDH (G8795), and anti-β-actin (Sigma A5441) overnight at 4°C. The membranes were washed three times in TBS-T and incubated with alkaline phosphatase conjugated secondary antibodies [Sigma A5153 (Mouse) and Sigma A9919 (Rabbit)] for 2 h at room temperature. Membranes were washed three times with TBST and developed using NBT-BCIP (Fermentes R0841, R821) in alkaline phosphatase buffer. After development of colored product on the membranes, they were washed with water, dried and images acquired using a scanner. Images were quantified using Image J software using β-actin and GAPDH as loading control for mice samples.

### Mass- Spectrometry

Heart tissue samples were homogenized using Rapigest SF Surfactant (Waters, #186001861), protein concentration was estimated and 100 ug of protein was used for sample preparation. 100 mM of freshly prepared 1, 4-dithiothreitol (DTT; Sigma #10197777001; final conc.10 mM) was added and incubated at 56°C for 40 min. The tubes were cooled to room temperature and 200 mM of freshly prepared Iodoacetamide (IAA, Sigma #I6125; final conc. 20 mM) was added and incubated for 1 h at room temperature. Excess IAA was quenched by adding DTT and incubated at room temperature for 20 min. Digestion was performed at 37°C overnight with mild agitation using 1 μg Trypsin protease (Pierce Thermo, #90057). The reaction was stopped the next morning using 0.1% Formic Acid (Sigma, #5330020050) and incubated at room temperature for 20 min. The samples were frozen, lyophilized and reconstituted in 0.1% formic acid before mass spectrometry analysis.

Reconstituted samples were subjected to mass spectrometric analysis using Orbitrap mass spectrometer (LTQ-XL, ThermoFisher Scientific) after chromatographic separation and peptide fractionation through a C18 easy spray nano column (3 μm, 100 A) by nano LC (Easy-nLC1000). The injection volume was 2 μl for each sample. Total gradient was set for 145 min with a spray rate of 300 nl/min. Scan range for Orbitrap was from 350 to 2,000 (m/z) with minimum three peaks and a resolution of 60,000. Ionic fragmentation was done by collision induced dissociation (CID) method. Protein identification was performed by Thermo Proteome Discoverer version 1.4.0. MS/MS spectra were matched against MASCOT in assistance with Percolator. Static Modification was set for N-terminal acetylation and Carbamidomethylation of Cysteine. Dynamic modification was set for Methionine (oxidation) and minimum missed cleavage number was set at two. Swissprot was used as reference software for protein identification.

Biological triplicates of the eight groups were compared using label-free relative quantification proteomic software SIEVE (Thermofisher Scientific, Waltham, MA, version.2.1.377). SIEVE directly processed the raw files from Thermo experimental datasets. To identify the statistically significant differences between LC-MS experiment datasets, the software calculated the *p*-value for the expression ratio of each differential peak. Spectra belonging to the peaks that were found to have statistically significant differential expression were then searched against the protein database MASCOT for peptide and protein identifications. Heat map was generated using graph pad prism software for the dataset.

PANTHER software (http://www.pantherdb.org/) was used for analyzing the significant alterations in the expressions of proteins and genes obtained from Thermo Proteome Discoverer and to identify their percent enrichment in Biological Processes and Molecular Functions.

The mass spectrometry proteomics data have been deposited to the ProteomeXchange Consortium *via* the PRIDE partner repository with the dataset identifier PXD025470.

Reviewer account details:

**Username:** reviewer_pxd025470@ebi.ac.uk

**Password:** z0EdUgUV.

### Cell Culture and Treatment

H9C2(2-1) cardiomyocytes were acquired from the National Center for Cell Science (Pune, India) and cultured in Dulbecco's modified Eagle medium (DMEM) with high glucose (4.5 g/liter), sodium bicarbonate (3.7 g/liter) and fetal bovine serum (FBS;10%) in an incubator maintained at 37 C, 5% CO_2_ and 80% relative humidity (RH). Cells were serum starved for 18–24 h before experimentation. Hypertrophy was induced by treating serum-starved H9C2 (2-1) cells with 100 μM Phenylephrine (PE; Sigma-Aldrich, USA) for 24h. PPAR α antagonist, GW 6471 (TOCRIS, 4618) dissolved in ethanol was used at a working concentration of 10 uM to block the PPAR α signaling.

### Autophagy Assay

H9C2 (2-1) cell line was seeded in a 6-well and divided into six groups. I-control, II-PE treated (treatment dose, 100 μM), III-serum starved, IV-GW6471 incubated (treatment dose, 10 μM), V-PE+GW6471, VI-serum starved+GW6471. After 24 h incubation, medium was removed 100 ul of autophagosome detection reagent working solution was added to each well as per manufacturer's instructions (Sigma-Aldrich, MAK138). After 15 min incubation at 37 C, 5% CO_2_ and 80% relative humidity (RH), each well was washed three times using PBS. Experiment was repeated along with two more groups I-cholesterol (50 μM), II-cholesterol+GW6471 to resemble the *in vivo* high cholesterol diet induced cardiac hypertrophy. Cells were seeded in a 96-well and same protocol was followed except the reading was taken using a microplate reader (Varioskan, LUX) at 518 nm.

### Proteome Profiler™ Antibody Array

Proteome profiler Antibody assay was performed using Proteome Profiler™ Antibody Array kit (R&D SYSTEMS) with 400 μg of protein per sample and the data obtained was quantified using Image J software.

### Echocardiography

The animal cardiac function was measured using the small animal imaging system (Vevo LAZR-X 3100, FUJIFILM Visual Sonics). Animals were anesthetized with 4% isoflurane and maintained anesthesia with 1–2% isoflurane during the Imaging procedure. Post-anesthesia, the animal was placed over the animal imaging platform. Essential parameters like respiration rate, electrocardiogram (ECG), body temperature, and heart rate were recorded throughout the imaging. The MX400 ultrasound transducer was used with a mouse small cardiology application in parasternal long-axis view (PLAX). Cardiac functions like cardiac output, ejection fraction, and fractional shortening were calculated from brightness mode (B-mode), where the heart left ventricular region was focused in a two-dimensional manner. Whereas Left ventricular mass (LV mass), systolic volume, diastolic volume were measured for each animal from the Motion Mode (M-mode) image. The echocardiography images were captured from videotape, and data were collected with the help of digital image analysis cardiac package software (Vevo Lab 3.1.1).

### Isolation and Culture of Neonatal Rat Ventricular Myocytes

Neonatal rat ventricular myocytes (NRVM) from 2-day-old Sprague-Dawley rat pups were isolated. Hearts were dissected and rinsed in Ads buffer (116.3 mMNaCl, 19.7 mM HEPES, 9.4 mMNaH2PO4, 5.5 mM glucose, 5.3 mMKCl, 0.83 mMMgSO4 [pH 7.4]). Atria were removed, and ventricles were minced and incubated at 37°C in a solution of enzymes containing 0.2% collagenase type II (381 U/mg) and pancreatin (0.6 mg/ml) in Ads buffer in the presence of 95% O2 and 5% CO2 for four successive digestions of 10 min each. The supernatant of the first tissue digestion was discarded. The supernatants obtained after each digestion were centrifuged for 5 min at 1,000 rpm. The cell pellets obtained in each round of digestion were resuspended in M199 growth medium (supplemented with 10% fetal bovine serum, and 100 U/ml penicillin-streptomycin, pH 7.2). The resuspended cells were pooled and passed through cell strainer, preplated in T25 cm^2^ flask (BD), and incubated for 1 h in a CO_2_ incubator for cardiomyocyte enrichment. The supernatants containing cardiomyocytes were collected by brief centrifugation and resuspended in growth medium. The cells were seeded onto collagen I-coated coverslips (Biocoat; BDLabware, Bedford, USA) in M199 medium supplemented with 10% FBS. Fresh media was replaced after 18 h of cell seeding and cultured for 2 days.

### Immunofluorescence and Microscopy

Cells were fixed in 4% paraformaldehyde for 20 min washed in PBS and incubated for 5 min in 0.2% triton X-100 in PBS. Cells were blocked in 1% BSA for 30 min before incubating with anti alpha -sarcomeric antibody (Sigma Aldrich) for overnight. Cells were washed two times in PBS for 5 min each and incubated with anti mouse Allexa flour 594 (molecular probes) for 1 h washed 3X and mounted on slide and pictured. High resolution image were acquired on 63x oil immersion objective lensNA 1.4,on Zeiss LSM 980 confocal microscope and processed on ZEN Blue. All images were acquired under identical Laser power, detector gain, offset and pinhole aperture window.

### Statistical Analysis

Statistical analysis was performed using Graph-Pad Prism Software version 6. Unpaired, 2-tailed Student's *t*-test or two-way ANOVA followed by *post-hoc* Tukey's Test was used to calculate statistically significant differences between control and experimental groups. Data represents mean ± S.D. of 3 individual experiments. Difference between two groups were analyzed using Student's t-test (control and experimental group), along with that individual experimental groups were also compared using Unpaired, 2-tailed Student's *t*-test. *p* < 0.05 was set as the threshold for statistical significance between the control and various experimental groups.

## Results

### Induction of Isoproterenol-Induced Cardiac Hypertrophy in PPAR α ^–/–^ Mice

**Figure 1:**
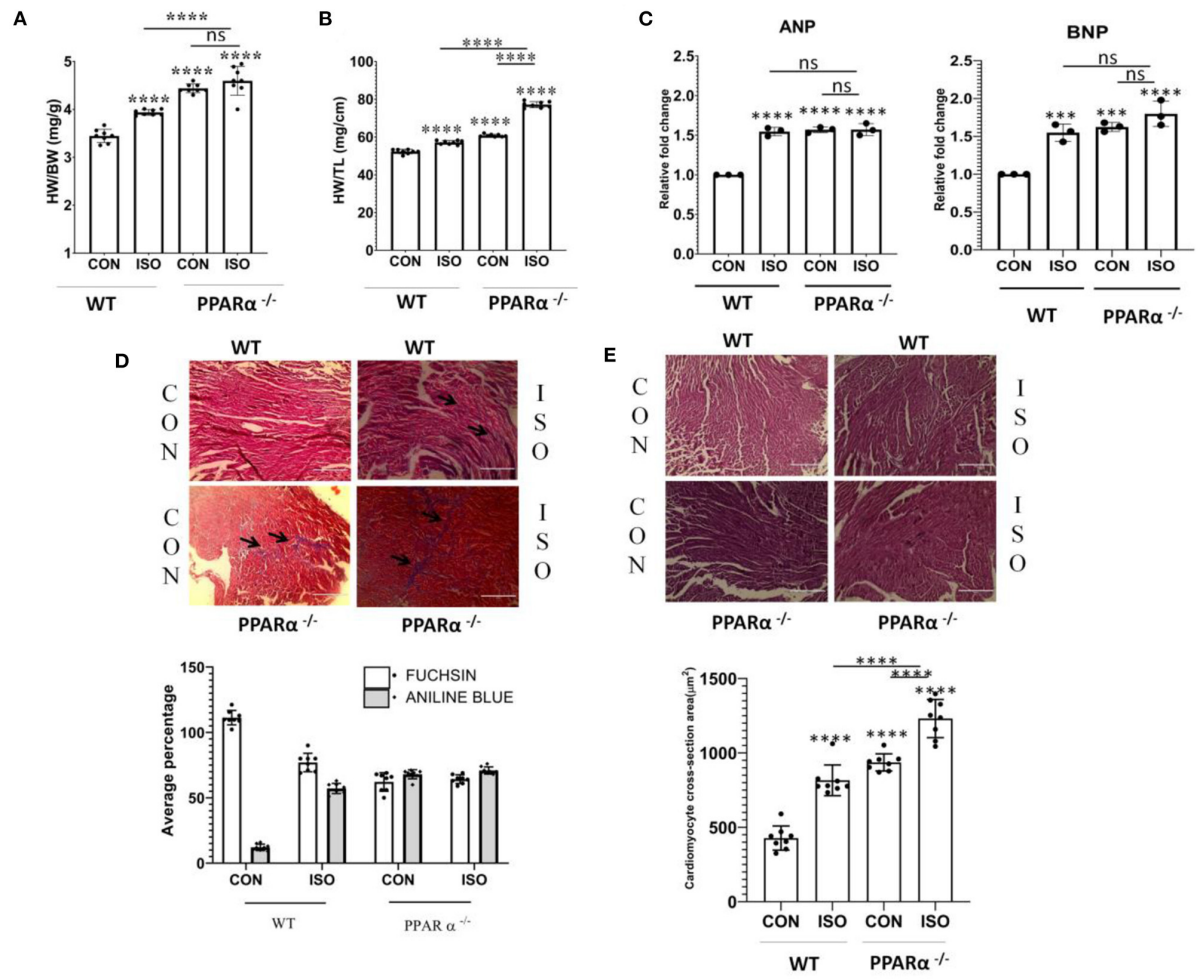
Development of cardiac hypertrophy in mice: C57BL/6 wild-type mice and PPAR α^**−/−**^mice (*N* = 8) were treated with or without isoproterenol (IP) for 14 days. Cardiac hypertrophy was examined by measuring heart weight to body weight ratio **(A)** as well as heart weight to tibia length ratio **(B)**. Gene expression of cardiac stress markers ANP and BNP were analyzed by real time PCR to assess hypertrophy **(C)**. GAPDH was used as a control for normalization in RT-PCR. Masson Trichome staining to detect collagen deposition; scale bar=200μm; (Blue=Fibrous collagen Red=myocytes, Black=Nuclei) and Bar graph representing statistically significant difference in collagen deposition (Aniline Blue) between C57BL/6 and PPAR α^**−/−**^ mice treated with or without isoproterenol **(D)**. Haematoxylin and Eosin (H & E) staining to detect morphological changes; scale bar = 200 μm Pink = Cytoplasmic protein, Blue/Purple = Nuclei and Bar Graph representing cardiomyocyte cross-section area (μm^2^) **(E)**. Data represents mean ± SEM of eight individual mice heart samples. Data was analyzed using two-way ANOVA with Tukey's *post-hoc* analysis. Difference between two groups was analyzed using Student's *t*-test. (***p < 0.001, ****p < 0.0001 vs. CON).

HW/BW ratio which is considered as the index of cardiac hypertrophy was monitored following 14 days treatment of PPAR α^−/−^ mice with β-adrenergic receptor agonist, isoproterenol (Figure 1A). The HW/BW ratio in PPAR α^−/−^ mice (4.45 ± 0.29 mg/g) was significantly increased (*p* > 0.0001) compared to that of wild-type mice (3.44 ± 0.13 mg/g, *p* > 0.0001) which was similarly enhanced (4.6 ± 0.29 mg/g, *p* > 0.0001) on isoproterenol treatment (Figure 1A). Thus, PPAR α^−/−^ mice developed cardiac hypertrophy (4.45 ± 0.299 mg/g, *p* > 0.0001), even without isoproterenol treatment. As shown in Figure 1A, hypertrophy was also induced in normal mice due to isoproterenol treatment (4.00 ± 0.059 mg/g, *p* > 0.0001). To further confirm the development of hypertrophy, the heart weight to tibia length (HW/TL) ratio was calculated in the same experimental animals. As shown in Figure 1B, HW/TL ratio was significantly increased due to isoproterenol treatment both in wild-type as well as PPAR α^−/−^ mice compared to the untreated animal.

### Fetal Gene Re-expression in Response to Isoproterenol Treatment

Cardiac hypertrophy is associated with cardiac remodeling, which brings about significant alterations in the cardiomyocytes including enhanced size, fibrosis and modification in transcriptional profile. One of such major changes is reverting back to fetal gene expression program. Atrial natriuretic peptide (ANP) and Brain natriuretic peptide (BNP) are specifically expressed in the heart in fetal stage and are switched off in the adult stage. Therefore, ANP and BNP are considered as markers of cardiac hypertrophy. Increased expression of ANP and BNP was observed both in wild type as well as PPAR α^−/−^mice in response to isoproterenol treatment (Figure 1C).

### Myocardial Fibrosis in Isoproterenol-Treated Wild-Type and PPAR α^–/–^ Mice

As cardiac hypertrophy results into myocardial fibrosis and increase in cardiomyocyte size, histological staining was carried out in heart tissue sections in both wild-type as well as PPAR α^−/−^ mice. Masson Trichome staining (Figure 1D) revealed collagen deposition in isoproterenol treated wild-type mice heart tissue sections when compared to untreated wild-type mice. Extensive collagen deposition pertaining to myocardial fibrosis was observed (Figure 1D) in isoproterenol-treated PPAR α^−/−^ mice heart tissue sections and similar results were observed in untreated PPAR α^−/−^ mice. Morphological examination using Haematoxylin and Eosin (H&E) staining revealed increased cardiomyocyte size in untreated PPAR α^−/−^ as well as isoproterenol-treated PPAR α^−/−^ mice (Figure 1E). The cardiomyocyte cross-section area (μm^2^) revealed enhanced cardiomyocyte size in wild-type isoproterenol treated mice heart tissue, along with untreated PPAR α^−/−^ as well as isoproterenol-treated PPAR α^−/−^ mice heart tissue when compared to untreated wild-type mice (Figure 1E).

### Echocardiographic Measurements Revealed Cardiac Dysfunction in PPAR α ^–/–^ Mice

**Figure 2:**
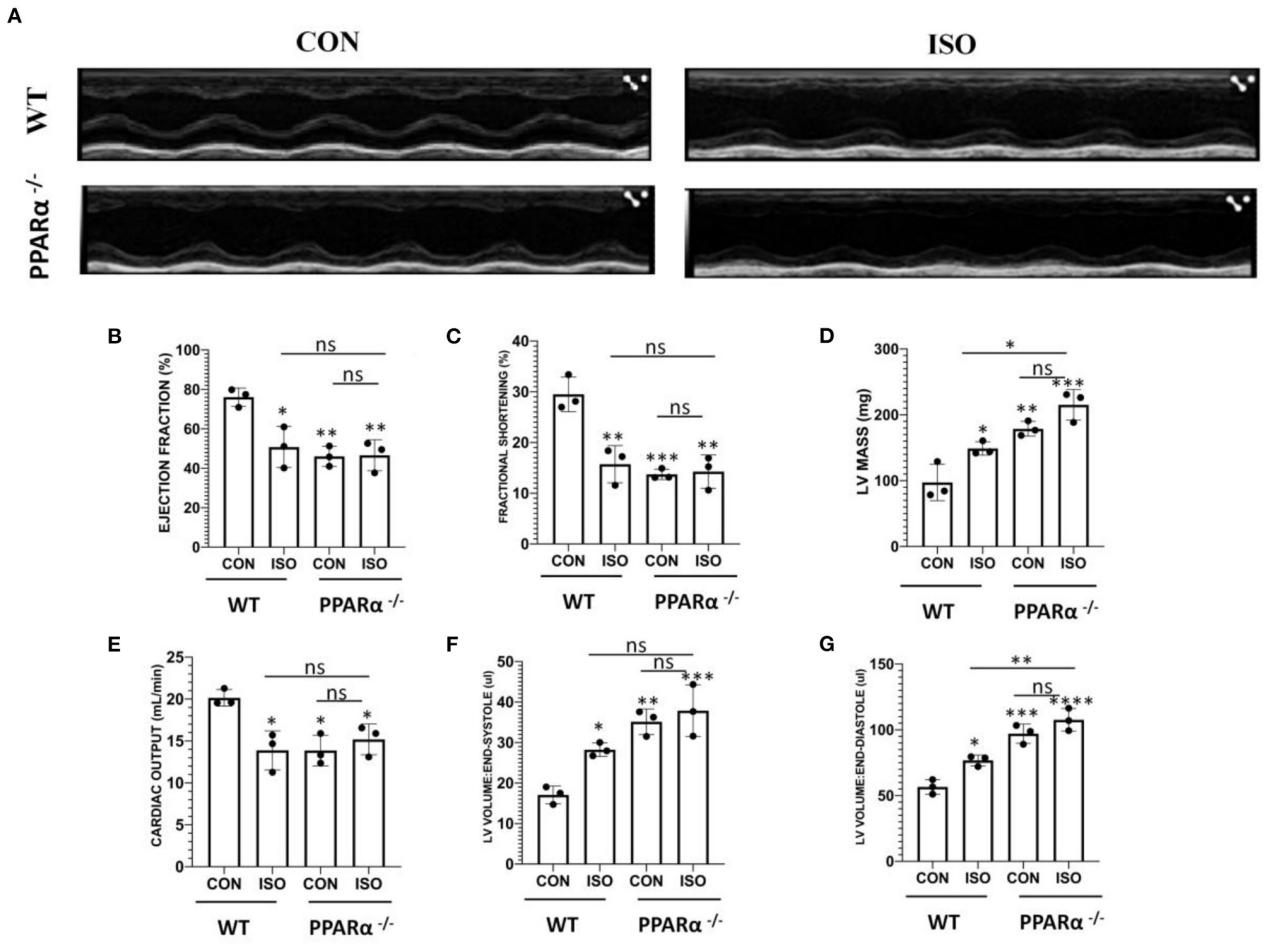
Echocardiography for *in vivo* assesment of cardiac function: Cardiac function was assessed by echocardiography in wild-type and PPAR α^−/−^ mice. Representative images from parasternal short axis view of the heart acquired in M-Mode **(A)**. Bar graphs showing ejection fraction **(B)**, fractional shortening **(C)**, LV mass **(D)**, cardiac output **(E)**, LV volume in end-systole **(F)** LV volume in end-diastole **(G)**. Data was analyzed using two-way ANOVA with Tukey's post-hoc analysis. (*p < 0.05, **p < 0.01, ***p < 0.001, ****p < 0.0001 vs. CON).

Cardiac function was assessed in wild-type and PPAR α^−/−^ mice by echocardiography following 14 day isoproterenol treatment. Representative echocardiographic images have been displayed in Figure 2A, that were obtained in M-Mode from each group. Chronic administration of isoproterenol resulted in significant impact on cardiac function in isoproterenol treated wild-type as well as PPAR α^−/−^ mice. Ejection fraction and fractional shortening were significantly decreased to a similar degree in isoproterenol-treated wild type, untreated PPAR α^−/−^ as well as isoproterenol-treated PPAR α^−/−^ mice. Ejection fraction was decreased by 33.29% in isoproterenol-treated wild type, 39.4% in untreated PPAR α^−/−^ mice and 38.73% in isoproterenol-treated PPAR α^−/−^ mice (Figure 2B). Fractional shortening was decreased by 46.7% in isoproterenol-treated wild type mice, 53.5% in untreated PPAR α^−/−^ mice and 51.67% in isoproterenol-treated PPAR α^−/−^ mice (Figure 2C). LV mass was enhanced significantly in isoproterenol-treated wild type mice, and was further escalated in untreated PPAR α^−/−^ as well as isoproterenol-treated PPAR α^−/−^ mice when compared to untreated wild-type mice (Figure 2D). Cardiac output was decreased by 31% both in isoproterenol-treated wild type and untreated PPAR α^−/−^ mice and 24.57% in isoproterenol-treated PPAR α^−/−^ mice (Figure 2E). End-systolic and diastolic volumes were significantly increased in isoproterenol-treated wild type mice (65.24 and 34.86%, respectively), untreated PPAR α^−/−^ mice (105.5 and 71.7%) and isoproterenol-treated PPAR α^−/−^ mice (121.5 and 90.4%) as shown in Figures 2F,G.

### Differential Protein Expression in Isoproterenol Treated PPAR α^–/–^ Mice Heart

**Figure 3:**
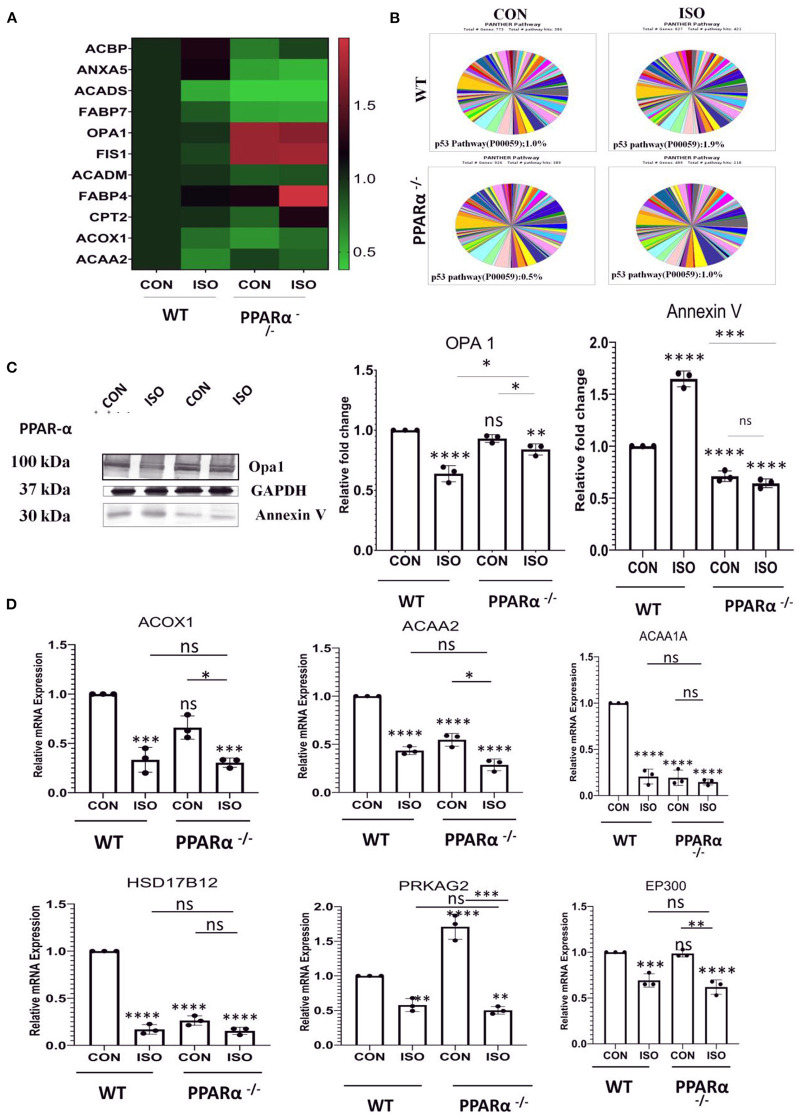
Identification of impaired signaling pathways in PPAR α^−/−^ mice heart by Proteomic analysis: Cardiac tissue proteins were analyzed by Orbitrap LC-MS (*N* = 5) followed by relative quantification of differentially expressed protein by SIEVE **(A)**. The validation of proteomic results for a subset of candidates was performed by RT-PCR using heart tissue obtained from the experiments as mentioned earlier. Expression of PPAR α target genes, such as ACOX1, Acaa1a, ACAA2, HSD17B12 along with genes independent of PPAR α regulation like PRKAG2 and EP300 were examined **(B)**. Enrichment of specific pathways were analyzed using Panther classification **(C)**. The expression of Annexin V and OPA 1 was examined by immunoblotting which was quantitated by densitometric analysis **(D)**. GAPDH was used as the loading control. Data represents mean ± S.D. of 3 separate experiments. Data was analyzed using two-way ANOVA with Tukey's *post-hoc* analysis. Difference between two groups was analyzed using Student's *t*-test. (*p < 0.05, **p < 0.01, ***p < 0.001, ****p < 0.0001 vs. CON).

To explore the pathways that are controlled *via* PPAR α in cardiac hypertrophy, we performed protein profiling in heart tissues collected from wild type and PPAR α^−/−^ mice by high resolution mass spectrometry. Differential protein expression pattern was observed, marked by an increase in mitochondrial fusion proteins like Opa1, fission protein FIS1 and down regulation of fatty acid binding protein like FABP7 (Figure 3A). PPAR α is a nuclear transcription factor which targets the genes involved in fatty acid β-oxidation and drives their transcription. Differential expression of PPAR α target genes as observed by proteomics analysis was also validated using RT-PCR. Fatty acid β-oxidation is a multi-step process and requires forefront play of multiple genes. Therefore, we examined several of its target genes in the heart tissue of the same mice. As shown in Figure 3B, the mRNA levels of Acox1, Acaa1a, Acaa2 and Hsd17B12 were decreased in isoproterenol treated PPAR α^−/−^ mice compared to wild type control. We also examined genes that are not transcriptionally regulated *via* PPAR α, but tend to alter in cardiac hypertrophy (PRKAG2 and EP300). As shown in Figure 3D, the expression of PRKAG2 and EP300 showed a significant decline in response to isoproterenol but no such down regulation was observed in the absence of PPAR α. Though PRKAG2 expression was down regulated in presence of isoproterenol, it was increased irrespective of PPAR α, whereas EP300 remained unaltered. The absence of PPAR α thus marks a reduction in fatty-acid β-oxidation *via* significant reduction in the genes that are essential in the process. As isoproterenol treatment in PPAR α^−/−^ mice exacerbates stress conditions that triggers apoptosis, we also examined some of the apoptotic markers that showed striking alteration in the proteome profile. Interestingly, SIEVE-based relative quantification revealed about 2-fold down regulation of Annexin V in PPAR α^−/−^ mice. In Isoproterenol-treated PPAR α^−/−^ mice, similar down regulation of Annexin V was observed. Panther classification of proteins by pathway analysis showed specific changes in cellular processes (Figure 3B). Interestingly, it revealed unexpected decrease in p53 pathway in the absence of PPAR α that is one of the inducers of apoptosis. Annexin V is a Ca^++^ dependent phospholipid binding protein, and actively binds phosphatidylserine, that is expressed on the outer leaflet of plasma membrane during apoptosis. Since most of the studies indicated the involvement of apoptosis in stress conditions, we further validated it *via* western blotting for AnnexinV, and observed reduced levels in the PPAR α^−/−^ as well as isoproterenol-treated PPAR α^−/−^ mice (Figure 3C). Western Blot was also performed for Opa1 to further validate the differential protein expression pattern. Opa1 expression was decreased on isoproterenol treatment in wild-type mice whereas normal level was observed in the absence of PPAR α (Figure 3C). Since hypertrophy associated cardiomyocyte apoptosis is a well-established phenomenon and our study revealed the down-regulation of Annexin V in isoproterenol treated PPAR α^−/−^ mice, we further examined other apoptotic markers in the same tissue.

### Down Regulation of Major Apoptotic Markers

**Figure 4:**
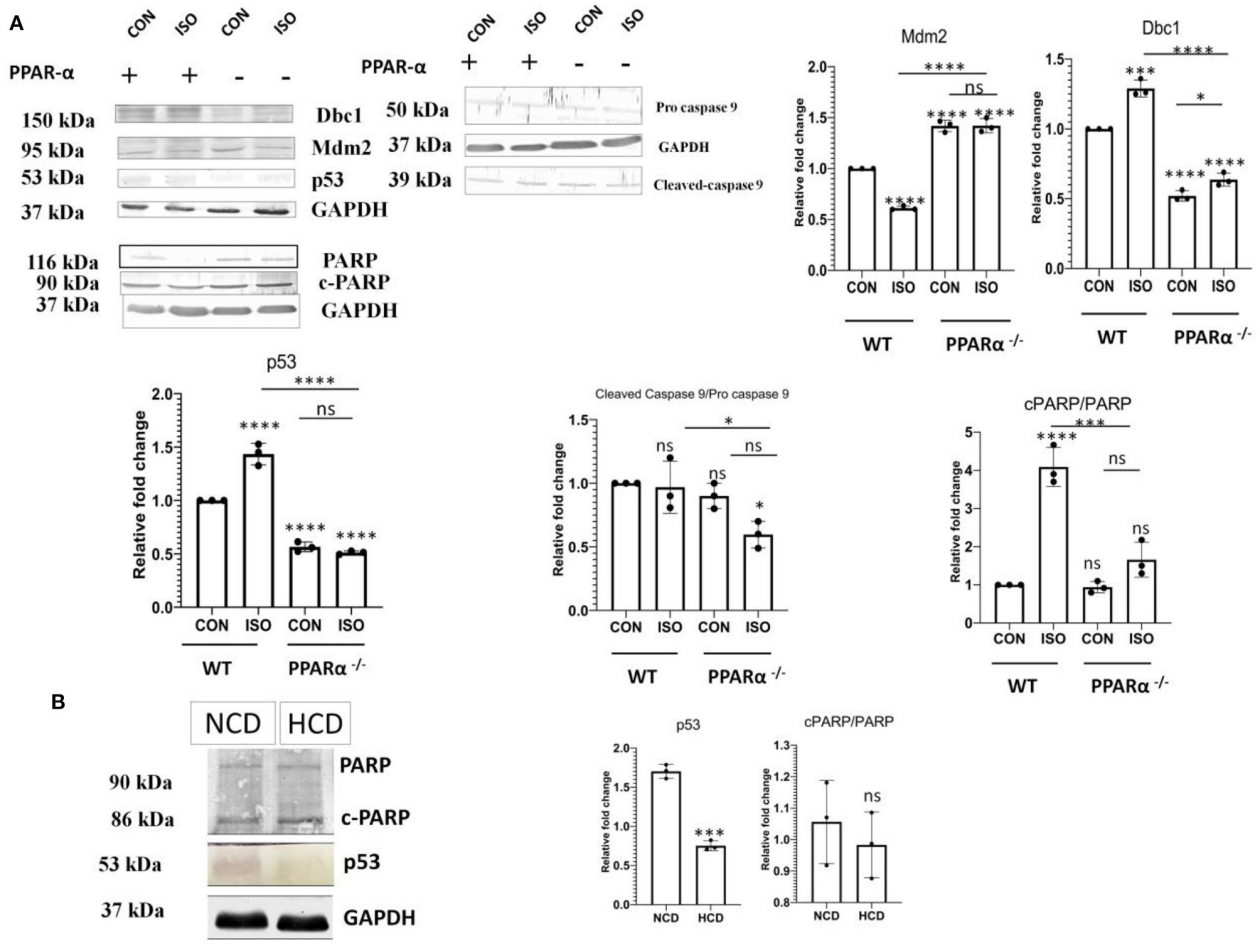
Attenuation of Apoptotic markers. Protein levels of apoptotic markers p53, Dbc1, Mdm2, c-caspase 9 and c-PARP in mice heart tissue obtained from the experiments as mentioned earlier were compared *via* immunoblot **(A)**. Protein levels of apoptotic markers p53 and c-PARP in normal chow diet fed (NCD) PPAR α ^**−/−**^ mice as well as high cholesterol diet fed (HCD) PPAR α ^**−/−**^ mice compared using immunoblot **(B)**. GAPDH was used as a loading control. Data represents mean ± S.D. of three individual experiments. Data was analyzed using two-way ANOVA with Tukey's *post-hoc* analysis. Difference between two groups was analyzed using Student's *t*-test. (*p < 0.05, ***p < 0.001, ****p < 0.0001 vs. CON).

Hypertrophy associated cardiac stress induces up regulation of critical apoptotic markers and directs the cell toward programmed cell death. In context to the previous results (Figure 3), we examined anti-apoptotic markers Bcl-2, Bcl-x, Catalase, HO-1, HO-2, Hsp70, Hsp27 and Hsp60 along with pro-apoptotic markers like Bad, Caspase 3, Claspin, Cytochrome C, FAS/CD95, Mcl-1, p53, p27/kip1, Smac/Diablo, TRAIL R2/TNFRSF10B, TNFR1/TNFRSF1A in wild-type controls as well as PPAR α^−/−^ mice, treated with isoproterenol using the proteome profiler (Supplementary Figure 2). Pro-apoptotic markers were down regulated in PPAR α^−/−^ mice whereas up regulation of anti-apoptotic markers was observed except Hsp27 as well as Hsp60, suggesting compensatory role of Hsp70 as all the three Heat shock proteins function to inhibit apoptosis. To elucidate whether this downregulation was limited to isoproterenol induced cardiac hypertrophy in PPAR α^−/−^ mice, we used high cholesterol diet fed PPAR α^−/−^ mice as another experimental model of pathological hypertrophy. Cardiac Hypertrophy in HCD fed PPAR α^−/−^ mice was confirmed *via* HW/BW ratio as well as HW/TL ratio (Supplementary Figure 1). Extensive collagen deposition and increased cardiomyocyte size further confirmed the development of cardiac hypertrophy in HCD fed PPAR α^−/−^ mice (Supplementary Figure 1). Significant down regulation of p53 and cleaved Caspase 9 was observed *via* immunoblotting analysis in PPAR α^−/−^ mice as well as isoproterenol-treated PPAR α^−/−^ mice when compared to wild-type control as well as isoproterenol-treated wild-type mice. Another apoptotic marker, c-PARP that is enhanced in isoproterenol-treated wild type mice, showed no change in PPAR α^−/−^ mice, but was significantly decreased in isoproterenol-treated PPAR α^−/−^ mice (Figure 4A). Similar results were observed for c-PARP as well as p53 protein in HCD fed PPAR α^−/−^ mice suggesting down regulation of apoptosis in the absence of PPAR α when stimulated with hypertrophy inducing factors (Figure 4B). Since caspase 9 is a key player in the formation of apoptosome that drives intrinsic apoptotic pathway, down regulation of cleaved caspase-9 suggests hindrance to intrinsic apoptotic pathway in the absence of PPAR α. So, it led us to further analyse the mediators of apoptosis.

### Regulation of p53 by PPAR α

It is established that p53 has a critical role in apoptosis and it binds to PPAR α. p53 is also known to be a positive regulator of fatty-acid β-oxidation. Therefore, we analyzed the fate of p53 in the absence of PPAR α. Isoproterenol-treatment of wild-type mice resulted in up regulation of p53, whereas the same treatment in PPAR α^−/−^ mice failed to escalate its levels (Figure 4A). Instead, there was a down regulation of p53 in PPAR α^−/−^ mice as well as in isoproterenol-treated PPAR α^−/−^ mice. Normally, p53 is very unstable but upon binding to Dbc1 its stability is improved. Dbc1 competes with Mdm2, a negative regulator, to promote p53 stability. Dbc1 expression was observed in PPAR α^−/−^ mice and it was found to be reduced along with p53 (Figure 4A). Thereby, suggesting a direct co-relation to PPAR α. To further understand the mechanism of p53 down regulation, we examined the levels of Mdm2. Mdm2 is known to negatively regulate p53 by preventing its physical binding to DNA as well as targeting it for proteasomal degradation. Mdm2 showed elevated levels in the absence of PPAR α (Figure 4A). Enhanced level of Mdm2 thereby explained the down regulation of p53. This data indicates that the p53-mediated apoptotic response was inhibited due to the absence of PPAR α.

### Modulation of PTEN in the Absence of PPARα

**Figure 5:**
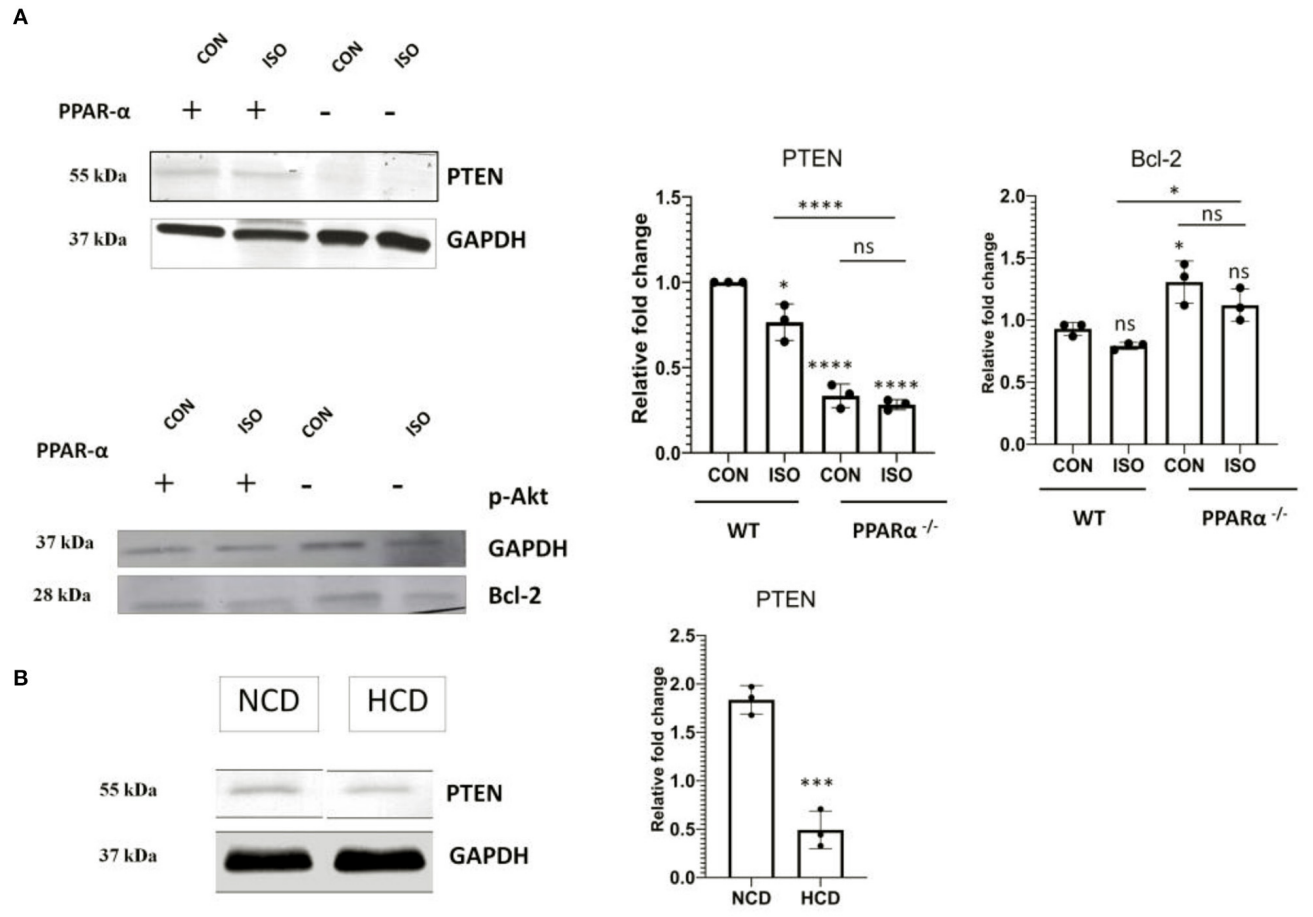
Modulation of PTEN: Protein level of apoptotic marker PTEN alongwith cell survival gene Bcl-2 was examined using immunoblot in heart tissue obtained from the experiments as mentioned earlier **(A)**. Protein level of PTEN in normal chow diet fed (NCD) PPAR α^**−/−**^ mice as well as high cholesterol diet fed (HCD) PPAR α^**−/−**^ mice compared using immunoblot **(B)**. GAPDH was used as a loading control. Data represent mean ± S.D. of three individual experiments. Data was analyzed using two-way ANOVA with Tukey's *post-hoc* analysis. Difference between two groups unpaired Student's *t*-test (*p < 0.05, ***p < 0.001, ****p < 0.0001 vs. CON).

PTEN that is an inducer of apoptosis in normal conditions and is transcriptionally activated *via* PPAR α was analyzed using western blot to understand the impact of PPAR α on apoptosis in cardiac hypertrophy. Down regulation of PTEN correlated with the absence of PPARα as observed in Figure 5A, whereas wild-type control showed normal PTEN level that was enhanced on isoproterenol treatment. Western Blot analysis of PTEN in PPAR α^−/−^ mice as well as High-Cholesterol Diet fed PPAR α^−/−^ mice showed similar pattern as hypertrophic stimulation in PPAR α^−/−^ mice failed to enhance PTEN levels (Figure 5B). Further downstream signaling proteins were analyzed like p-Akt and mTOR as well as pro survival protein Bcl-2, all of which showed upregulation in the absence of PPAR α. This suggested an inclination toward survival upon hypertrophic stimulation as observed by the enhanced protein levels in PPAR α^−/−^ as well as isoproterenol-treated PPAR α^−/−^ mice when compared to wild-type control as well as isoproterenol-treated wild-type mice (Supplementary Figure 3). Similar results were obtained in high cholesterol diet fed PPAR α^−/−^ mice wherein enhanced levels of mTOR was observed *via* immunoblotting, although no significant change was observed in pAkt protein levels between normal chow diet fed PPAR α^−/−^ mice and HCD fed PPAR α^−/−^ mice (Supplementary Figure 3).

### Up Regulation of Autophagy Markers

**Figure 6:**
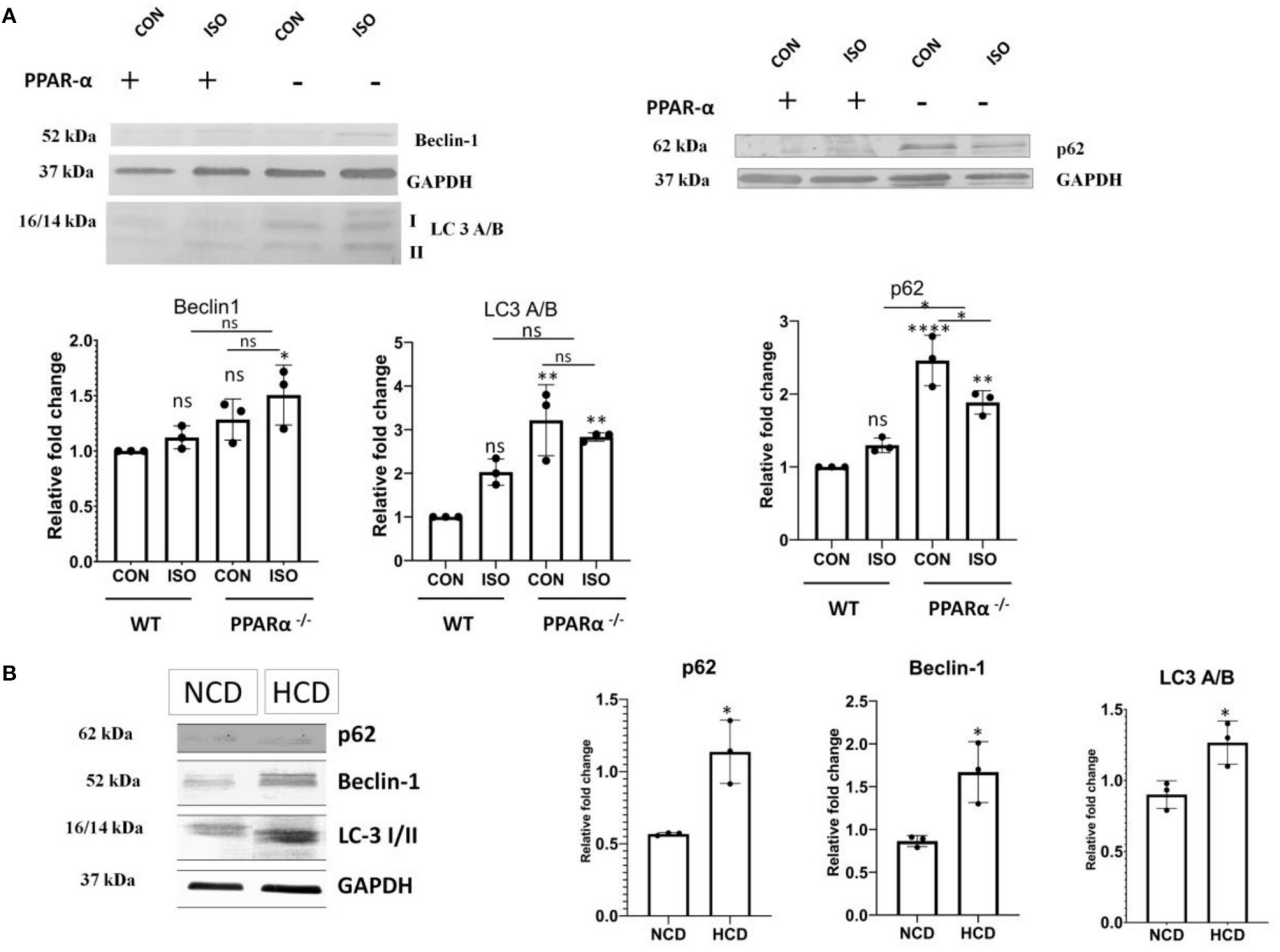
Upregulation of autophagy markers in PPAR α^−/−^mice: Autophagy markers p62, Beclin-1 and LC3 A/B were examined using western blot and densitometric analysis **(A)**. p62, Beclin-1 and LC3 A/B were analyzed in normal chow diet fed (NCD) PPAR α^**−/−**^ mice as well as high cholesterol diet fed (HCD) PPAR α^**−/−**^ mice using immunoblot **(B)**. GAPDH was used as a loading control. Data represent mean ± S.D. of three individual experiments. Data was analyzed using two-way ANOVA with Tukey's *post-hoc* analysis. Difference between two groups was analyzed using Student's *t*-test (*p < 0.05, **p < 0.01, ****p < 0.0001 vs. CON).

Autophagy is negatively regulated by mTOR and the down regulation of apoptosis indicated the switching on a compensatory mechanism for managing the stress-induced cardiomyocyte. Autophagy is known to play essential role in quality control through clearance of damaged proteins and organelles. Therefore, we examined the level of autophagy markers. As shown in Figures 6A,B, the levels of the autophagy markers such as p62, Beclin1 and LC3 A/B showed a significant increase in heart tissue of PPAR α^−/−^ mice when compared to control (*p* < 0.001). Both PPAR α^−/−^ as well as isoproterenol-treated PPAR α^−/−^ mice showed an upregulation when compared to wild-type control as well as isoproterenol-treated wild-type mice indicating an induction of autophagy and an inclination toward adaptive response that might help in sustaining the cardiac stress (Figure 6A). HCD fed PPAR α^−/−^ mice showed similar increased levels of autophagy markers p62, Beclin1 and LC3A/B when compared to normal chow diet fed PPAR α^−/−^ mice indicating an upsurge toward autophagy in PPAR α^−/−^ mice upon hypertrophic stimulation.

### Upregulation of Atg 3, 5 and 7 in the Absence of PPAR α

**Figure 7:**
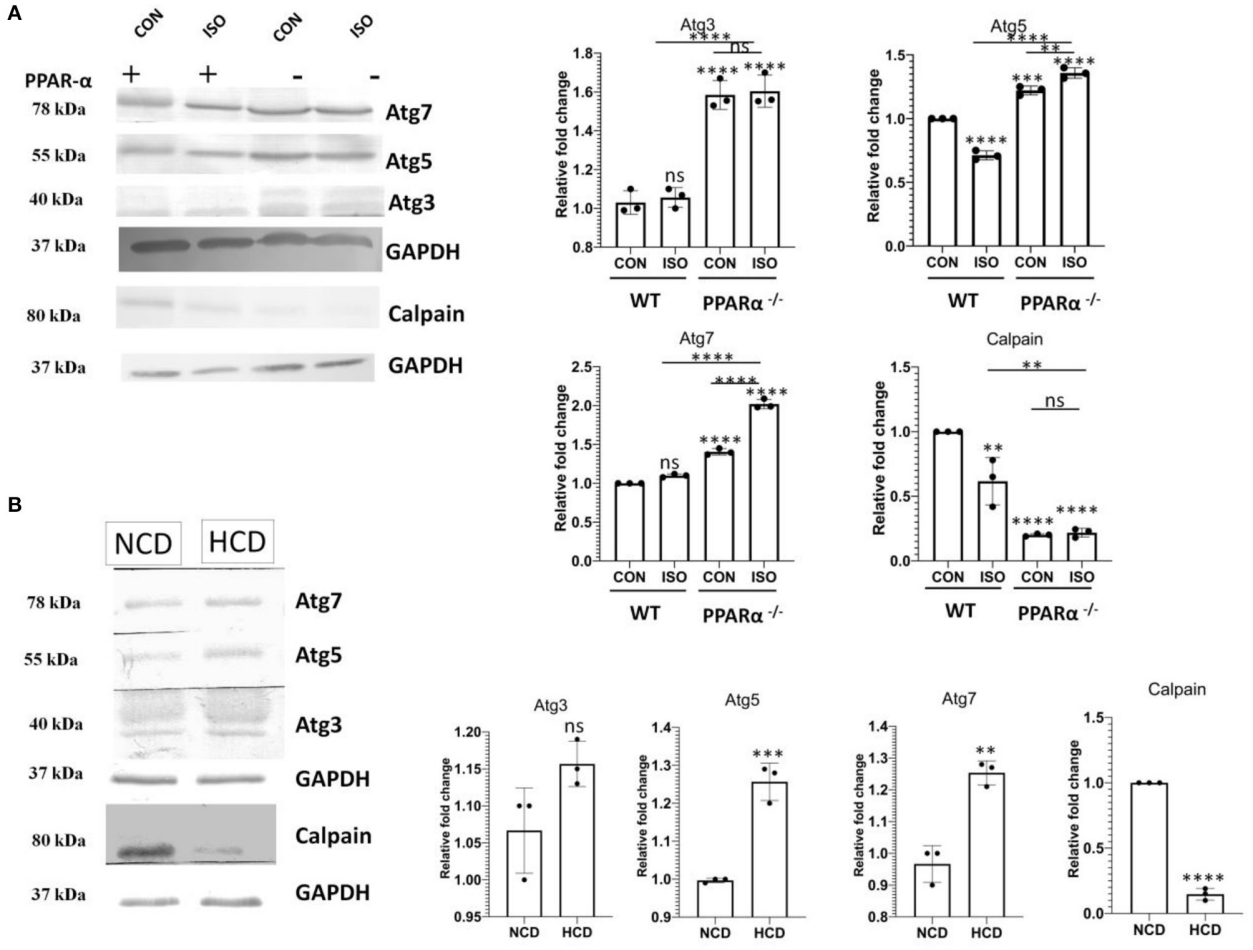
Downregulation of Calpain and upsurge of Atg genes in PPAR α^−/−^ mice: Atg 3, Atg 5 and Atg7 along with Calpain were examined using western blot and densitometric analysis **(A)**. Expression of Atg 3, Atg 5, Atg 7 and Calpain was analyzed in normal chow diet fed (NCD) PPAR α^**−/−**^ mice as well as high cholesterol diet fed (HCD) PPAR α^**−/−**^ mice using immunoblot **(B)**. GAPDH was used as a loading control. Data represent mean ± S.D. of three individual experiments. Data was analyzed using two-way ANOVA with Tukey's *post-hoc* analysis. Difference between two groups was analyzed using Student's *t*-test (**p < 0.01, ***p < 0.001, ****p < 0.0001 vs. CON).

Atg genes that are known to mediate autophagy were analyzed using western Blot. Atg 3, 5 and 7 in PPAR α^−/−^ as well as isoproterenol-treated PPAR α^−/−^ mice were found to be upregulated when compared to wild-type control as well as isoproterenol-treated wild-type mice indicating an enhanced autophagy even in the presence of mTOR (Figure 7A). Moreover, HCD fed PPAR α^−/−^ mice resembled this pattern of Atg genes when compared to NCD fed PPAR α^−/−^ mice, thus Atg genes are substantially increased in PPAR α^−/−^ mice specifically when stimulated with hypertrophy inducing factor (Figure 7B). To further understand this autophagy induction even in the presence of mTOR, calpain protein levels were analyzed. Calpain is an inhibitor of autophagy and its downregulation mediates mTOR independent autophagy. Significant decrease in the protein levels of calpain in both the experimental models explained the increase in autophagy in a mTOR-independent manner (Figures 7A,B).

### Downregulation of PTEN and Induction of Autophagy in Cardiomyocytes *in vitro*

**Figure 8:**
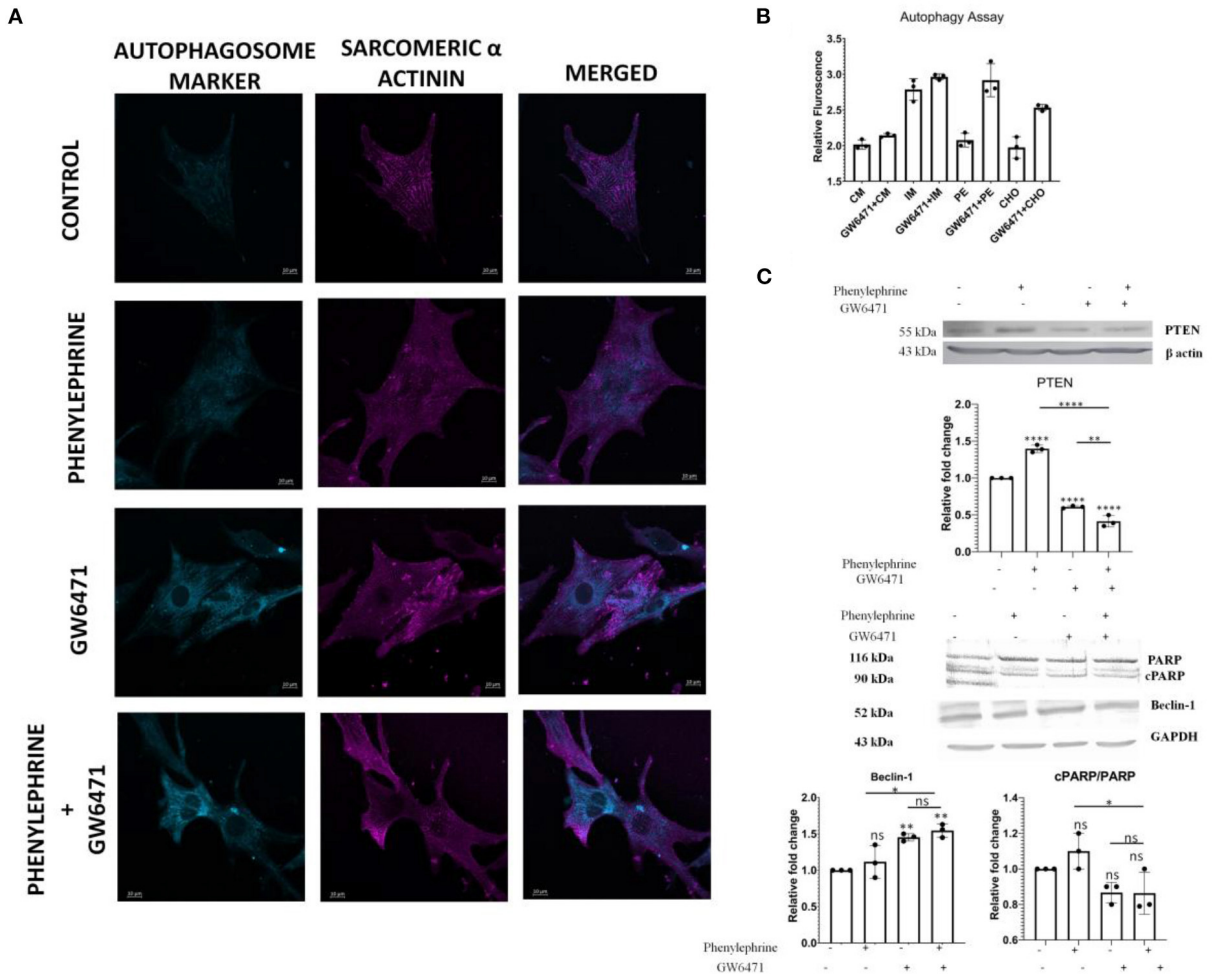
Enhanced Autophagy and alteration of apoptosis and autophagy markers in H9C2 cell line: Representative Confocal images of Autophagy Assay in NRVMs of different treatment groups; scale bar = 10 μm **(A)**. Bar Graph depicting the relative fluorescence in the indicated experiments in H9C2 cell line **(B)**. Western Blot analysis and the quantitative results of PTEN, cPARP/ PARP and Beclin1 **(C)**. Data represent mean ± S.D. of 3 individual experiments. α-actin was used as a loading control. Data was analyzed using two-way ANOVA with Tukey's *post-hoc* analysis. Difference between two groups was analyzed using Student's *t*-test (*p < 0.05, **p < 0.01, ****p < 0.0001 vs. CON).

Our previous observations demonstrated the downregulation of apoptosis followed by an increase in autophagy in hypertrophy induced in PPAR α^−/−^ mice. Next, we further investigated the effects of PPAR α absence upon hypertrophic stimulation *in vitro*. Neonatal rat ventricular myocytes (NRVM) were treated with phenylephrine (PE) a β-adrenergic receptor agonist for 48 h and PPAR α was inhibited using GW6471, PPAR α antagonist. Consistent with previous results, autophagy assay revealed enhanced autophagy in GW6471 incubated myocytes, even in the presence of PE when compared to control as was indicated by an elevated number of bright blue dot stained autophagic vacuoles (Figure 8A). Confocal microscopy revealed sarcomeric striation pattern through α-actinin staining (pink) along with the autophagic vacuoles (Figure 8A). PE alone failed to show such increase in autophagy, suggesting that hypertrophy in the absence of PPAR α antagonist, shows no such inclination toward autophagy as the absence of PPAR α mediates this alteration. Measurement of relative fluorescence performed in H9C2 cardiomyocytes using microplate reader revealed similarly enhanced autophagy when PPAR α was blocked by GW6471. Also, cholesterol incubated cells in the presence of GW6471 showed increase in autophagy when compared to cells treated with cholesterol alone (Figure 8B). To elucidate the status of apoptosis *in vitro*, PTEN expression was analyzed in H9C2 cells which was found to be increased in the presence of PE which was significantly down regulated in the presence of GW6471.Autophagy marker Beclin1 was checked *in vitro* and the results somewhat resembled *in vivo* data and showed slight increase in comparison to untreated control cells, whereas apoptosis marker cPARP showed no change between the experimental groups (Figure 8C).

## Discussion

The myocardium responds to mechanical as well neurohumoral stimuli by adjusting myocytes size accompanied with intracellular molecular switching for maintaining normal functions (24–26). However, persistence of hypertrophic conditions may lead to maladaptation and cardiac failure (27–29). Such maladaptation is partly due to the failure of the intracellular machineries for coping up to the altered hemodynamics (30). Impaired PGC 1 α – PPAR α signaling, mitochondrial dysfunctions and imbalanced bioenergetics are known to contribute significantly to this maladaptation of hypertrophic myocardium (31–33). It has been observed in previous studies that hypertrophy- induced post–translational modification of PPAR α causes a decline in the ligand sensitivity and expression of PPAR α, thus is directly associated with the impaired fatty acid oxidation (5). This could be suggestive of the fact that cardiomyocytes undergo certain adaptive modifications to meet up with the increase in the energy demand in response to hypertrophic stimuli. Here we show that PPAR α plays a central role in the adaptive phase of the cardiomyocytes by driving toward autophagy and preventing apoptosis. We desired to study these alterations in the cardiac cells for which, we used isoproterenol to develop cardiac hypertrophy in PPAR α^−/−^ mice model.

Intra – peritoneal injection of isoproterenol for duration of 2 weeks resulted in the development of hypertrophied hearts in the PPAR α^−/−^ mice as evident from HW/BW ratio and the HW/TL ratio. It is observed that hypertrophy in isoproterenol-treated wild-type mice and isoproterenol-treated PPAR α^−/−^ mice were more pronounced when compared to the control (untreated wild type). Additionally, the development of hypertrophy is validated by increased expression of hypertrophic marker genes such as ANP and BNP. Histopathological study revealed increased collagen deposition indicating extensive fibrosis in the absence of PPAR α which is the hallmark of cardiac remodeling. The absence of PPAR α thereby seems to be associated with myocardial fibrosis that tends to be an outcome of the stress in its absence.

Consistent with hypertrophic pathophysiology, the PPAR α^−/−^ mice show significant alterations in heart functions which are more pronounced due to treatment with isoproterenol. Reduction in Ejection fraction as well as Fractional shortening occurred in both the isoproterenol-treated groups. In the absence of isoproterenol, these parameters are compromised in PPAR α^−/−^ mice suggesting that absence of PPAR α is enough to cause cardiac dysfunctions. Evidently, cardiac output showed marked decline in all the groups when compared to wild-type control further affirming to the impaired heart functions in hypertrophied heart. Increased LV mass further confirmed the development of cardiac hypertrophy in the mice model.

We used the protein profiling data generated from the mass spectrometric analysis of the heart tissue to examine the proteins that, in deficiency of PPAR α, are closely associated with cardiac stress and are involved, especially, in mitochondrial dysfunction and impaired fatty acid β-oxidation pathway. A marked increase in the mitochondrial fusion protein like Opa1, fission protein FIS1 while a decrease in the levels of FABP7—a fatty acid binding protein, ACAA1A, ACOX1 and several such critical genes involved in the Fatty acid β-oxidation were observed, thereby reflecting the impact of absence of PPAR α in distinct signaling pathways. Validation of the mass spectrometric data through both immunoblot as well as qpcr, revealed a similar expression pattern. One of the striking changes observed was the decline in apoptotic pathway. Surprisingly, SIEVE-based relative quantification, as well as western blot analysis, revealed about a significant down regulation of Annexin V (an apoptotic marker) in both the isoproterenol—treated and untreated PPAR α ^−/−^ mice unlike what has been reported in previous studies. Isoproterenol-induced cardiac hypertrophy has been related with increased stress, that exhibits cardiac remodeling whereas in this scenario the results indicated an abrupt change in p53-induced apoptotic pathway in the absence of PPAR α, that was therefore looked further into detail to understand the mechanism.

The earlier studies related the involvement of apoptosis in the pathogenesis of cardiac remodeling (12, 34–36). As treatment of PPAR α^−/−^ mice with isoproterenol aggravates stress conditions, it is likely that apoptosis is triggered under those conditions. So, to confirm the observed data, proteome profiler Antibody Array was used to assess multiple genes regulating apoptosis. Several anti-apoptotic genes like Bcl-2, Bcl-x, HO-1 were observed to be either up regulated or showing no change in PPAR α^−/−^ mice and isoproterenol-treated PPAR α^−/−^ mice, suggesting moderate inclination toward survival while most of the pro apoptotic genes like Bad, Caspase 3, Claspin, Cytochrome C, FAS/CD95, Mcl-1, p53, p27/kip1, Smac/Diablo, TRAIL R2/TNFRSF10B, TNFR1/TNFRSF1A showed down regulation in the PPAR α^−/−^ as well as isoproterenol treated PPAR α^−/−^ mice when compared to normal wild-type mice. Isoproterenol-treated wild-type mice as expected revealed upsurge in the apoptotic markers exhibiting the impact of isoproterenol-induced cardiac remodeling that initiates stress conditions resulting in cardiomyocyte death. Therefore, the absence of PPAR α leads to perturbation of the genes involved in the apoptotic pathway in the heart as observed from the proteome profiler data, where unlike wild-type isoproterenol treated mice there was no increase in apoptotic markers level. Thus, we wished to validate the same proteome profile data of the apoptotic markers in our test model using western blot.

To further confirm this phenomenon is dependent on PPAR α, another experimental model was used, that is high-cholesterol diet fed PPAR α ^−/−^ mice model, wherein the implicated pathological cardiac hypertrophy was independent of isoproterenol treatment. Previous reports have already suggested the involvement of high cholesterol diet in inducing pathological cardiac hypertrophy but the study has been mostly limited to wild-type mice and its impact on PPAR α ^−/−^ mice is yet to be explored. Therefore, in our study PPAR α ^−/−^ mice fed with high-cholesterol diet for 8 weeks were used, wherein the cardiac hypertrophy was established and the hypertrophic markers like ANP and BNP also showed an upsurge as expected, apart from these, the Masson Trichome staining revealed significant accumulation of collagen in both PPAR α^−/−^ mice as well as HCD fed PPAR α^−/−^ mice.

Apoptotic markers were further assessed using immunoblot in both the experimental models. Interestingly, western blot analysis revealed a significant reduction in Caspase-9 in the absence of PPAR α whereas no significant changes were found in c-PARP protein levels. Since Caspase 9 is one of the key components of the intrinsic apoptotic pathway (37, 38), its down regulation implies possible interference in the basic apoptotic signaling induced by the deficiency of PPAR α as similar results were observed in both the cardiac hypertrophy models. Further analysis of the mediators of apoptosis using Panther based classifications divulged a surprising decrease in p53 pathway in the absence of PPAR α. It is a well-known fact that p53 is an inducer of stress-activated apoptosis (39–41). The recent discovery of the binding potential of p53 to PPAR α and its role as a positive regulator of fatty-acid β-oxidation, led us to analyze the fate of p53 in PPAR α^−/−^ mice (42). A significant downregulation was observed in both normal and hypertrophied PPAR α^−/−^ mice in contrast to the up-regulation in WT mice (treated as well as control). To confirm a direct relationship between p53 and PPAR α, we examined the expression profile of Dbc1 and Mdm2. Previous reports have shown the importance of Dbc1 in p53 stabilization by competing with Mdm2, a negative regulator of p53 stability (43, 44). Therefore, it is likely that p53 downregulation might be a result of the decline in the expression of Dbc1 and an elevation in Mdm2 expression. In support of our hypothesis Dbc1 expression was found to be reduced in PPAR α^−/−^ mice along with p53, in contrast, to an increase in Mdm2 expression. Thus, PPAR α could be suggested to hinder p53-mediated apoptotic response. Both PPAR α^−/−^mice as well as isoproterenol-treated PPAR α^−/−^mice along with the second experimental model i.e. PPAR α^−/−^mice as well as HCD fed PPAR α^−/−^mice showed similar pattern of the down regulation of the apoptotic markers thereby suggesting the involvement of PPAR α.

To investigate the role of PPAR α in down regulation of apoptosis in the cardiomyocytes and the underlying mechanism, the PTEN/pAkt/mTOR pathway was dissected. PTEN has emerged as a major regulator of apoptosis and many reports have suggested the critical role of PTEN in modulating apoptosis, apart from the previous studies that have also addressed the PPAR α dependent transcriptional regulation of PTEN (45). Western blot analysis of PTEN revealed down regulation in PPAR α ^−/−^, mice both in the response to isoproterenol as well as HCD thereby indicating a causative role of PPAR α with the down regulation of PTEN. Since PTEN/pAkt/mTOR pathway is a conglomeration of different signaling network that involves, cell survival, protein synthesis and regulation of apoptosis, so pAkt as well as mTOR were also analyzed using western blot to reveal the preference of cardiomyocytes toward survival when apoptosis is compromised. There was no significant change in pAkt but the up regulation of mTOR in PPAR α^−/−^ mice could be explained by the nutrient rich condition in the absence of PPAR α due to the accumulation of fatty acids as Fatty acid β-oxidation was significantly affected.

Since many reports have addressed the regulation of autophagy *via* mTOR, so the autophagy pathway was analyzed in PPAR α^−/−^mice. The significant increase in mTOR suggested a parallel down regulation of autophagy. Surprisingly, various autophagy markers like p62, Beclin1 and LC3 A/B revealed significant up regulation in the absence of PPAR α expression. The increase in p62 could be explained as the role of p62 is subjective to the cell type. The increase in autophagic flux in the heart, results in concurrent upregulation of p62 that plays a protective role in aggresome formation and in the activation of autophagy in stress-induced cardiomyocytes. Since mTOR negatively regulates autophagy, so mTOR-independent pathway supposedly maintains an upsurge in the autophagy markers. To further confirm this, the protein of level of Calpain-1, that is a renowned mTOR independent modulator of autophagy was analyzed, and reduction in Calpain in the absence of PPAR α explains the increase in autophagy that does not correlate with the increase in mTOR (46). Significant increase in Autophagy genes like Atg3, Atg 5, Atg7, and Atg12 were observed in both isoproterenol-treated as well as high cholesterol diet fed PPAR α ^−/−^ mice. The data explains although the phenomenon is PPAR α dependent but is exhibited mostly in the presence of a hypertrophic signal.

To further validate the down regulation in apoptosis followed by an upsurge in autophagy in cardiomyocytes in the absence of PPAR α, experiments were also carried out in H9C2 cell line. GW6471 was used to block PPAR α and cardiac hypertrophy was induced using phenylephrine that is a well-known inducer of cardiac hypertrophy (47). Western blot analysis of PTEN resembled the similar pattern as observed *in vitro* thereby indicating PPAR α dependent phenomenon. Increase in autophagy marker Beclin1 was observed in PE-treated H9C2 cell line in the absence of PPAR α whereas apoptotic marker cPARP showed no significant change. To further validate the increase in autophagy, autophagosome assay was performed in H9C2 cell line, wherein serum starved cardiomyocytes that were used as positive control for autophagy were compared with PE treated as well as GW6471 incubated cell. Elevation of autophagosome formation was observed in the presence of GW6471 when compared to PE treated cells, indicating the enhanced autophagy as observed *in vivo* in the absence of PPAR α. Cholesterol treated H9C2 cell line in the presence of GW6471 showed significant increase in autophagy when compared to cells incubated with cholesterol alone, thereby indicating the absence of PPAR α might function as pre-requisite to mediate such change in signaling.

Few pieces of evidence reveal the presence of an association between cardiac hypertrophy and autophagy of the cardiomyocytes (48–50). With the down regulation of apoptosis, it could be suggested that to maintain cardiac homeostasis the autophagic flux might play a compensatory role in stress-induced cardiomyocytes. The increase in autophagic markers like Atg3, Atg5, and Atg 7 in the absence PPAR α expression might drive the cardiomyocytes toward autophagy as compared to apoptosis due to cardiac stress, as an adaptive measure.

The absence of PPAR α is associated with various pathophysiological changes including cardiac remodeling and hypertrophy, however, in the presence of hypertrophy inducing signal, PPAR α tends to promote apoptosis that eventually leads to cardiomyocyte death, whereas its absence causes a shift toward autophagy rather than apoptosis even in the presence of hypertrophic signals. Such adaptive shift as evident from increase in autophagy markers possibly explains the importance of the compensatory mechanism that operates in the cardiomyocytes in order to survive the myocardial stress through baseline autophagy.

**Figure 9:**
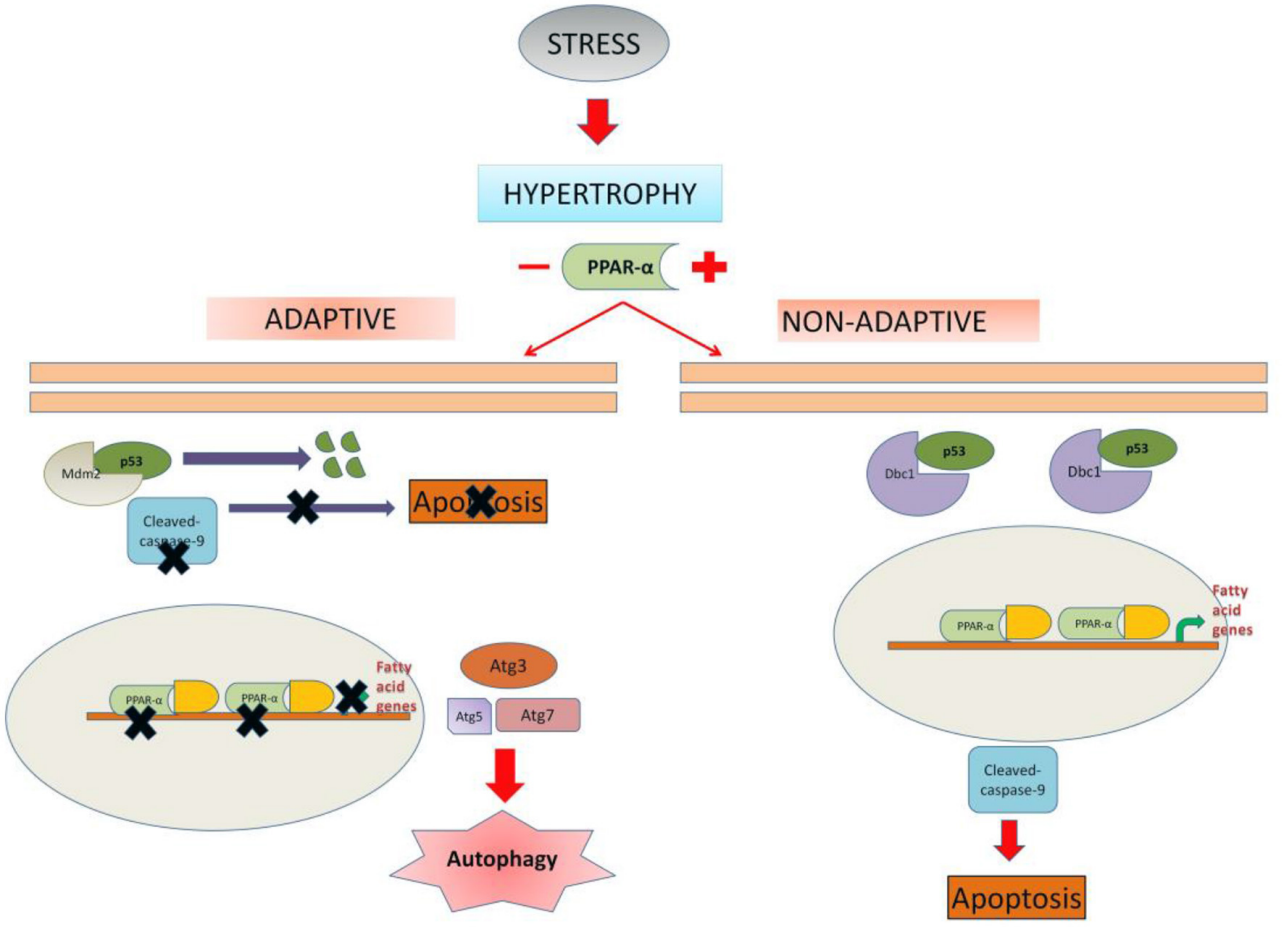
Schematic representation depicting the possible mechanism of cell fate regulation by PPAR α: In unregulated hypertrophy, PPAR α is likely to promote apoptosis of the cardiomyocytes leading to dysfunctional state of the myocardium. In the absence of PPAR α, cardiomyocytes are driven toward autophagy which appears to be an adaptive exercise. Therefore, down regulation of PPAR α during hypertrophy is likely to assist the cardiomyocytes in the compensatory mechanism.

In summary, although PPAR α is known to be downregulated in cardiac hypertrophy, this study unveiled a new mechanistic significance of it in the adaptation of the cardiomyocytes which is especially important in the compensatory phase. In normal situation PPAR α drives toward apoptosis whereas, in the absence of PPAR α, hypertrophic cardiomyocytes are driven toward autophagy. It is likely that hypertrophic cardiomyocytes are protected by switching autophagy which might act as a protective mechanism in the early adaptive phase of myocyte hypertrophy (Figure 9).

## Data Availability Statement

The datasets presented in this study can be found in online repositories. The names of the repository/repositories and accession number(s) can be found below: http://www.proteomexchange.org/, PXD025470. Raw Data for western Blot has been added as Supplementary Figure 5.

## Ethics Statement

The protocol was approved by the CSIR-IICB Animal Ethics Committee (Reference no. IICB/AEC/Meeting/Sep/2019/4) and the animals were handled in accordance with the Committee for the Purpose of Control and Supervision of Experiments on Animals (CPCSEA), Ministry of Social Justice, and Government of India (Registration no. 147/1999/CPCSEA).

## Author Contributions

RK and AR designed and performed the experiments, analyzed results, and drafted the manuscript. DM, VC, UK, DB, SB, DK, and AK conducted experiments. AB analyzed the data, arranged resources, and edited the manuscript. All authors contributed to the article and approved the submitted version.

## Funding

This work is supported by grant (MLP-115) from CSIR-Indian Institute of Chemical Biology, Kolkata. RK is a recipient of Senior Research Fellowship from CSIR, New Delhi, India.

## Conflict of Interest

The authors declare that the research was conducted in the absence of any commercial or financial relationships that could be construed as a potential conflict of interest.

## Publisher's Note

All claims expressed in this article are solely those of the authors and do not necessarily represent those of their affiliated organizations, or those of the publisher, the editors and the reviewers. Any product that may be evaluated in this article, or claim that may be made by its manufacturer, is not guaranteed or endorsed by the publisher.

## Supplementary Material

Supplementary Figure 1 Development of cardiac hypertrophy in High-cholesterol diet fed PPAR α-/-mice.

Supplementary Figure 2 Proteome profiler Antibody assay in PPAR α-/-mice.

Supplementary Figure 3 Upregulation of survival markers in PPAR α-/-mice.

Supplementary Figure 4 Genotyping of PPAR α-/-mice.

Supplementary Figure 5 Raw images of western Blot.

## Abbreviations

PPARα: Peroxisome proliferator-activated receptor alpha
PGC 1 α: PPAR γ (Peroxisome proliferator-activated receptor gamma) coactivator 1 alpha
ACOX1: Acyl-CoA Oxidase 1
Acaa1a: Acetyl-CoA Acyltransferase 1
ACAA2: acetyl-Coenzyme A acyltransferase 2
HSD17B12: 17-beta hydroxysteroid dehydrogenase-12
PRKAG2: Protein Kinase AMP-Activated Non-Catalytic Subunit Gamma 2
EP300: E1A Binding Protein P300
GAPDH: Glyceraldehyde 3-phosphate dehydrogenase
ANP: Atrial Natriuretic peptide
BNP: Brain Natriuretic peptide
LC 3 I/II: Microtubule-associated protein light chain 3
Dbc1: Deleted in bladder cancer protein 1
Mdm2: Mouse double minute 2 homolog
FIS 1: Mitochondrial fission 1 protein
FABP7: Fatty acid binding protein 7
OPA1: Optic Atrophy 1
c-PARP: cleaved-Poly (ADP-ribose) polymerase
WT: wild-type.
